# Mycobacterium tuberculosis Beijing Lineage and Risk for Tuberculosis in Child Household Contacts, Peru

**DOI:** 10.3201/eid2603.191314

**Published:** 2020-03

**Authors:** Chuan-Chin Huang, Alexander L. Chu, Mercedes C. Becerra, Jerome T. Galea, Roger Calderón, Carmen Contreras, Rosa Yataco, Zibiao Zhang, Leonid Lecca, Megan B. Murray

**Affiliations:** Brigham and Women’s Hospital, Boston, Massachusetts, USA (C.-C. Huang, M.C. Becerra, Z. Zhang, M.B. Murray);; Harvard Medical School, Boston (C.-C. Huang, M.C. Becerra, M.B. Murray);; Harvard University Division of Continuing Education, Cambridge, Massachusetts, USA (A.L. Chu);; University of South Florida, Tampa, Florida, USA (J.T. Galea);; Socios En Salud Sucursal, Lima, Peru (R. Calderon, C. Contreras, R. Yataco, L. Lecca)

**Keywords:** Tuberculosis, infection transmission, latent tuberculosis infection, BCG vaccine, Mycobacterium tuberculosis, tuberculosis and other mycobacteria, bacteria, Peru

## Abstract

Few studies have prospectively compared the relative transmissibility and propensity to cause disease of *Mycobacterium tuberculosis* Beijing strains with other human-adapted strains of the *M. tuberculosis* complex. We assessed the effect of Beijing strains on the risk for *M. tuberculosis* infection and disease progression in 9,151 household contacts of 2,223 culture-positive pulmonary tuberculosis (TB) patients in Lima, Peru. Child contacts exposed to Beijing strains were more likely than child contacts exposed to non-Beijing strains to be infected at baseline, by 12 months of follow-up, and during follow-up. We noted an increased but nonsignificant tendency for child contacts to develop TB. Beijing strains were not associated with TB in adult contacts. These findings suggest that Beijing strains are more transmissible in children than are non-Beijing strains.

Tuberculosis (TB) remains the leading cause of death worldwide from a single infectious disease; in 2017, ≈10 million incident cases and 1.7 million deaths were reported (*1*). The causative pathogen, *Mycobacterium tuberculosis*, is divided into 7 human-adapted phylogenetic lineages, of which some are geographically restricted and others are widespread throughout the world, possibly because they are better adapted to environments of high human density (*2*). One of the widespread groups, lineage 2, includes a widely distributed genotype, the Beijing strain, that has been repeatedly implicated in outbreaks and in the evolution of drug resistance (*3**–**6*). The global distribution of Beijing strains and their precipitous rise in some populations have led researchers to speculate that it may be more transmissible and more likely to cause disease than other less widely distributed *M. tuberculosis* lineages (*7**–**9*).

However, the few direct assessments of the relative transmissibility of Beijing strains have been inconclusive. Although some studies found that exposure to Beijing strains was more likely than exposure to other strains to lead to TB (*10**–**15*), others reported no difference (*16**,**17*). Several studies have suggested that the Beijing genotype is more common among young persons and that its frequency declines with age (*18**–**21*). To explore these factors, we directly compared the relative transmissibility and propensity to cause disease of Beijing strains with other strains in a cohort study of household contacts of patients with pulmonary TB in Lima, Peru.

## Methods

### Ethics Statement

The Institutional Review Board of Harvard School of Public Health and the Research Ethics Committee of the National Institute of Health of Peru approved the study. All study participants or their guardians provided written informed consent, and children <18 years of age provided assent.

### Setting, Study Design, and Participant Recruitment and Follow-Up

The study design and methods were previously described in detail (*22*). In brief, we conducted a prospective cohort study of household contacts of pulmonary TB patients in Lima, Peru. The study area comprised 20 districts inhabited by ≈3.3 million residents living in urban areas and in periurban, informal shantytown settlements.

During September 2009–August 2012, we identified patients >15 years of age who had received a diagnosis of clinically presumptive pulmonary TB at any of 106 participating health centers. We confirmed the microbiological status of their disease with either a positive sputum smear or mycobacterial culture. We also recorded the index patient’s sociodemographic data; baseline smear status; presence or absence of cavitary disease; tobacco and alcohol use; HIV status; time from symptom onset until initiation of treatment; and, for patients, with drug-resistant TB, the time from TB diagnosis until start of an effective treatment (i.e., a drug regimen deemed appropriate for the index patient’s drug-resistant profile).

Within 2 weeks after index patient diagnosis, we enrolled all consenting household contacts. We assessed baseline *M. tuberculosis* infection status with the tuberculin skin test (TST) in household contacts with no history of a positive TST or TB. Household contacts who had signs or symptoms of TB underwent clinical evaluation and, if indicated, initiated treatment under Peru's National Tuberculosis Program guidelines (*23*). We offered HIV testing to all study participants. In accordance with Peru’s National Tuberculosis Program guidelines, isoniazid preventive therapy (IPT) was offered to household contacts <19 years of age and persons with specified concurrent conditions. At the time of household contact enrollment, we collected age, sex, sociodemographic, and occupational information; height and weight; alcohol and tobacco use information; HIV status; self-reported diabetes mellitus; history and presence of *M. bovis* BCG vaccination scars; use of IPT; history of TB; and housing features. We repeated the TST 6 and 12 months after initial evaluation among household contacts who had previously tested negative; 2, 6, and 12 months after initial evaluation, we reevaluated them for pulmonary and extrapulmonary TB. We also accessed the medical records of health centers in the catchment areas to identify household contacts whose TB was diagnosed at a health center during the follow-up period.

### Whole-Genome Sequencing

Among 3,027 index patients with culture-positive isolates at baseline, 2,143 had isolates that also underwent whole-genome sequencing (WGS) using the Illumina HiSeq 4000 platform (Illumina, https://www.illumina.com) with a read length of 100–150 bp and >50-fold coverage. The raw sequence data were trimmed using the sickle package (*24*) and mapped to the H37Rv reference genome using the BWA-MEM algorithm (*25*). We used a coverage-based approach, specifically SAMtools (default settings) (*26*) and pilon (*27*), to identify single-nucleotide polymorphisms across the whole genome. We assigned a call as missing if the valid depth of coverage at a specific site was <8 reads, if the mean read-mapping quality at the site did not reach 9, or if none of the alternative alleles accounted for >85% of the valid coverage.

### Lineage Identification of *M. tuberculosis* Strains

Among index patients for whom WGS data were available, we identified *M. tuberculosis* lineages and sublineages on the basis of a previously published barcode that differentiates the groups on the basis of single-nucleotide polymorphisms (*28*). For index patients without available WGS data, we genotyped their *M. tuberculosis* isolates using 24-locus mycobacterial interspersed repetitive units–variable-number tandem repeats (MIRU-VNTR) (*29*). We used the best-match algorithm from the MIRU-VNTR*plus* online database (http://www.miru-vntrplus.org) to identify the lineages of the typed samples by cross-referencing them with reference lineages stored on the database (*30*).

### Outcomes

We considered 4 distinct outcomes: *M. tuberculosis* infection at baseline, time to TST conversion during the 12 months of follow-up, *M. tuberculosis* infection by 12 months of follow-up, and incident disease during the 12-month follow-up time. We classified household contacts as infected at baseline if they reported a history of TB, reported a previous positive TST result within 6 months after study enrollment, or had a positive TST (induration size >10 mm [>5 mm for HIV-positive household contacts]) at baseline. We considered initially TST-negative household contacts to have undergone TST conversion if their TST status converted from negative to positive or if TB developed during follow-up. We considered household contacts to be infected with *M. tuberculosis* by 12 months of follow-up if contacts were infected at baseline or had a TST conversion during the 12-month follow-up period (*22*). We considered household contacts to have co-prevalent TB if it was diagnosed within 2 weeks after the diagnosis in the index patient. If household contacts received diagnoses 2 weeks–15 months (1-year follow-up plus a 3-month buffer period) after index patient diagnosis, we considered incident TB to have developed in those contacts during follow-up. We based TB diagnosis among contacts >18 years of age on the same criteria we used for index patients; the diagnosis among household contacts <18 years was based on published consensus guidelines from an expert panel on classifying TB in children (*31*).

### Statistical Analysis

We made an a priori decision to analyze child household contacts (<15 years) and adult household contacts (>15 years) separately for several reasons. First, we considered child contacts to be a more representative age group for household-based TB transmission than their more mobile, socially active adult counterparts. Second, several previous studies suggest that Beijing strains more commonly affect children than adults (*18**–**20*). We used complete data analyses in all multivariate adjustment models and conducted all statistical analyses in R version 3.5.1 (https://www.r-project.org).

#### TB at Baseline and by 12 Months of Follow-Up

We used a modified Poisson generalized estimating equation to measure the association between exposure to index patients infected with Beijing strains and the likelihood of infection at baseline and by 12-months of follow-up. To account for correlation within households, we specified an exchangeable working correlation structure for observations within the same household and obtained empirical SE estimates for robust inference. We constructed univariate and multivariate models to compare the effect of exposure to Beijing and non-Beijing strains on the risk for TB infection at baseline and at 12 months. In the multivariate models, we included covariates from index patients (age, drug resistance profile, and HIV status) and their household contacts (age, diabetes status, BCG vaccination status, socioeconomic status, and nutritional status). We conducted sensitivity analyses in which we excluded household contacts who reported a history of TB, a previous positive TST result within 6 months after study enrollment, or a history of receiving TB treatment.

#### Time to TST Conversion and to Incident Disease

We assessed the time to TST conversion among household contacts who were uninfected at baseline by defining the date of infection as the midpoint between the date of enrollment and the date of a first positive TST result or TB diagnosis. We excluded from analysis household contacts who remained TST negative at the date of their last TST result. For the time to TB analysis, we excluded household contacts with co-prevalent TB and excluded from analysis household contacts in whom TB had not been diagnosed by the time of their death or at the end of the study. We first plotted Kaplan-Meier survival curves to visually evaluate the association between the index patient’s *M. tuberculosis* genotype and the 2 time-to-event outcomes. We then used Cox frailty proportional hazards model to evaluate risk factors for time to TST conversion and time to incident disease while accounting for clustering within households. We constructed univariate and multivariate models to compare the effect of exposure to Beijing and non-Beijing strains on the risk for TST conversion and incident disease during follow-up. In the multivariate models, we included covariates from index patients (age, drug resistance profile, and HIV status) and their household contacts (age, diabetes status, BCG vaccination status, socioeconomic status, and nutritional status). We further adjusted for the use of IPT and TB history in the multivariate model of the time to incident disease analyses. We verified the proportional hazards assumptions for each covariate by including an interaction term for the covariate and time and stratifying by covariates for which the assumption did not hold. We conducted 2 sensitivity analyses, 1 in which we defined TST conversion using an increment of >6-mm induration size in repeat TST measurements during follow-up and 1 in which we restricted the analysis to household contacts in whom active TB developed during follow-up and who received their diagnoses within >30 days after index patient enrollment.

#### Effect of Index Patient *M. tuberculosis* Lineage on Protective Efficacy of BCG Vaccination

We also considered the possibility that index patient *M. tuberculosis* lineage might modify the protective efficacy of BCG vaccine. To examine whether index patient *M. tuberculosis* strain type modified the association between BCG vaccination and outcomes of our study, we added in our model an interaction term that included index patient *M. tuberculosis* lineage and the BCG vaccination status of household contacts.

## Results

We enrolled 9,151 household contacts of 2,223 culture-positive pulmonary TB index patients. The study enrollment rates were 85.5% for index patients and 94.6% for their household contacts. The retention rates for enrolled household contacts were 92.0% at 6 months and 94.7% at 12 months of follow-up. At baseline, 841 (27.0%) of 3,115 child household contacts and 3,007 (53.1%) of 5,663 adult household contacts were infected. A total of 951 (43%) index patients had lineage 4.1 strain isolates, 775 (35%) had lineage 4.3 strain isolates, 255 (12%) had lineage 2 isolates (all of which were of the Beijing strain), and 242 (11%) had other strain isolates (Table 1). We determined the baseline characteristics for household contacts, stratified by age at <15 and >15 years (Table 2; Appendix Tables 1–4).

**Table 1:** Baseline characteristics of *Mycobacterium tuberculosis* culture-positive index patients, Lima, Peru, September 2009–August 2012

Variable	No. (%), N = 2,223*
Age, y	
16–30	1,363 (61)
31–45	465 (21)
>46	395 (18)
Sex	
M	1,289 (58)
F	934 (42)
Concurrent condition	
HIV seropositive	59 (3)
Self-reported diabetes	111 (6)
Current smoker	60 (3)
*M. tuberculosis* lineage	
L2 (Beijing)	255 (12)
L4.1	951 (43)
L4.3	775 (35)
Other	242 (11)
Sputum smear status†	
Negative	548 (25)
+	639 (29)
++	431 (19)
+++	596 (27)
Cavitary disease	655 (30)
Drug resistance profile	
Pansusceptible	1,442 (67)
Resistant	726 (33)

*Numbers might not add to total because of missing data. †+, 1–99 acid-fast bacilli (AFB) in 100 observed fields; ++, 1–10 AFB per field in 50 observed fields; +++, >10 AFB per field in 20 observed fields.

**Table 2:** Baseline characteristics of household contacts exposed to a *Mycobacterium tuberculosis* culture-positive index tuberculosis patient, Lima, Peru, September 2009–August 2012

Variable	Total no. (%), N = 9,151*	Age <15 y, no. (%)	Age >15 y, no. (%)
Age, y			
<15	3,225 (35)	3,225 (100)	Not applicable
>15	5,926 (65)	Not applicable	5,926 (100)
Sex			
M	4,147 (45)	1,620 (50)	2,527 (43)
F	5,004 (55)	1,605 (50)	3,399 (53)
Concurrent condition			
HIV seropositive	33 (<1)	3 (<1)	30 (1)
Self-reported diabetes	165 (2)	2 (<1)	163 (3)
Current smoker	559 (6)	4 (<1)	555 (10)
*M. tuberculosis* lineage exposure			
L2 (Beijing)	1,041 (11)	349 (11)	692 (12)
L4.1	3,733 (41)	1,332 (41)	2,401 (41)
L4.3	3,416 (37)	1,202 (37)	2,214 (37)
Other	961 (11)	342 (11)	619 (10)
Presence of *M. bovis* BCG vaccination scar	8,110 (89)	2,876 (89)	5,253 (89)
Nutritional status			
Normal weight	5,261 (58)	2,527 (79)	2,734 (47)
Underweight	163 (2)	95 (3)	68 (1)
Overweight	3,636 (40)	565 (18)	3,071 (52)
Socioeconomic status			
Low	3,112 (35)	1,253 (40)	1,859 (33)
Middle	3,991 (45)	1,384 (44)	2,607 (45)
High	1,828 (21)	514 (16)	1,314 (23)
Isoniazid preventive therapy recipient	2,090 (23)	1,542 (48)	490 (7)

*Numbers might not add to total because of missing data.

### *M. tuberculosis* Infection at Baseline and After 12 Months of Follow-up

Child contacts exposed to Beijing strains were more likely than those exposed to non-Beijing strains to be infected at baseline (risk ratio [RR] 1.27 [95% CI 1.06–1.53]; adjusted RR [aRR] 1.23 [95% CI 1.02–1.50]) (Table 3) and by 12 months of follow-up (RR 1.20 [95% CI 1.08–1.35]; aRR 1.22 [95% CI 1.09-1.38]) (Table 4). The prevalence of *M. tuberculosis* infection at baseline (RR 0.99 [95% CI 0.91–1.08]; aRR 1.00 [95% CI 0.91–1.09]) and by 12 months (RR 1.00 [95% CI 0.96–1.05]; aRR 1.01 [95% CI 0.97–1.06]) did not vary by index patient *M. tuberculosis* strain in adult household contacts (Tables 3, 4). In the sensitivity analyses, we obtained almost identical results when we excluded household contacts who reported a history of TB, a previous positive TST result within 6 months after study enrollment, or a history of TB treatment (Appendix Tables 5, 6).

**Table 3:** Effect of the *Mycobacterium tuberculosis* Beijing lineage on the risk for infection at baseline in child and adult household contacts of culture-positive index patients, Lima, Peru, September 2009–August 2012

Lineage	Age <15 y, n = 3,115*				Age >15 y, n = 5,663*		
	Prevalence, no. (%)†	Risk ratio (95% CI)			Prevalence, no. (%)†	Risk ratio (95% CI)	
		Univariate	Multivariate‡			Univariate	Multivariate‡
Non-Beijing	731 (26)	Referent	Referent		2,654 (53)	Referent	Referent
Beijing	110 (33)	1.27 (1.06–1.53)	1.23 (1.02–1.50)		353 (53)	0.99 (0.91–1.08)	1.00 (0.91–1.09)

*Likelihood ratio test for the interaction between age (**<**15 or >15) and index patient *M. tuberculosis* lineage: p = 0.025. †Prevalence of baseline *M. tuberculosis* infection in the univariate model. ‡Multivariate model adjusted for index patient drug resistance profile; index patient age (16–30, 31–45, 46–60, and >60 y); index patient HIV status; household contact age (0–5, 6–10, 11–15, 16–30, 31–45, 46–60, and >60 y); household contact *M. bovis* BCG vaccination status; household contact socioeconomic status; household contact nutritional status.

**Table 4:** Effect of the *Mycobacterium tuberculosis* Beijing lineage on the risk for infection by 12 months of follow-up in child and adult household contacts of culture-positive index patients, Lima, Peru, September 2009–August 2012

Lineage	Age <15 y, n = 2,521*				Age >15 y, n = 4,921*		
	Prevalence, no. (%)†	Risk ratio (95% CI)			Prevalence, no. (%)†	Risk ratio (95% CI)	
		Univariate	Multivariate‡			Univariate	Multivariate‡
Non-Beijing	1,098 (49)	Referent	Referent		3,546 (82)	Referent	Referent
Beijing	173 (59)	1.20 (1.08–1.35)	1.22 (1.09–1.38)		474 (82)	1.00 (0.96–1.05)	1.01 (0.97–1.06)

*Likelihood ratio test for the interaction between age (<15 or >15 y) and index patient *M. tuberculosis* lineage: p = 0.003. †Prevalence of *M. tuberculosis* infection by 12 months for the univariate model. ‡Adjusted for index patient drug resistance profile; index patient age (16–30, 31–45, 46–60, and >60 y); index patient HIV status; household contact age (0–5, 6–10, 11–15, 16–30, 31–45, 46–60, and >60 y); household contact *M. bovis* BCG vaccination status; household contact socioeconomic status; household contact nutritional status.

### TST Conversion

**Figure 1:**
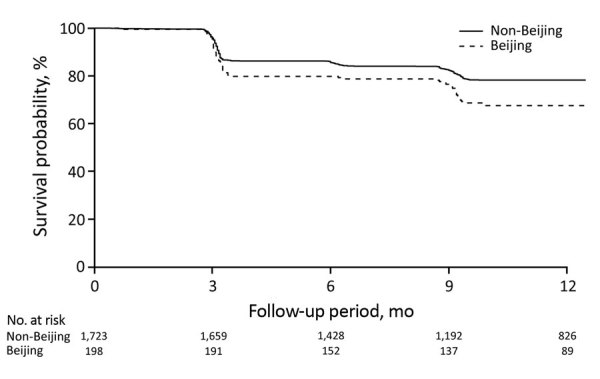
Survival curves for incident *Mycobacterium tuberculosis* infection in child household contacts by index patient *M. tuberculosis* lineage, Lima, Peru, September 2009–August 2012.

**Figure 2:**
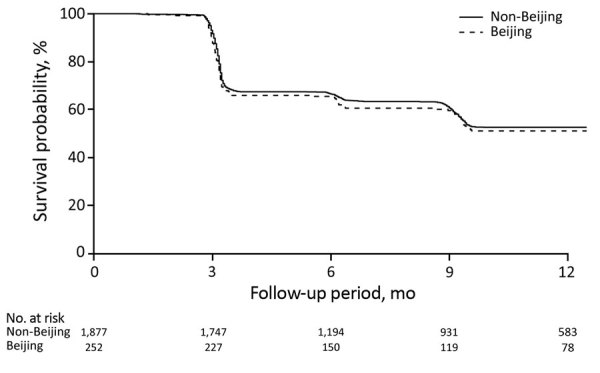
Survival curves for incident *Mycobacterium tuberculosis* infection in adult household contacts by index patient *M. tuberculosis* lineage, Lima, Peru, September 2009–August 2012.

The TST status of children exposed to Beijing strains were more likely than that of children exposed to other strains to convert from negative to positive during 12 months of follow-up in both the univariate and multivariate analyses (hazard ratio [HR] 1.53 [95% CI 1.09–2.14]; adjusted HR [aHR] 1.65 [95% CI 1.17–2.33]). Exposure to Beijing strains had no differential effect in adults (HR 1.02 [95% CI 0.80–1.30]; aHR 1.03 [95% CI 0.80–1.33]) (Table 5; Figures 1, 2). In the sensitivity analyses, we defined TST conversion by an increment of >6 mm in TST induration size during follow-up, and the results changed by <5% (Appendix Table 7).

**Table 5:** Hazard ratios of tuberculin skin test conversion comparing contacts exposed to a *Mycobacterium tuberculosis* Beijing lineage with those exposed to a non-Beijing lineage, Lima, Peru, September 2009–August 2012

Lineage	Age <15 y*				Age >15 y*		
	Incidence, %†	Hazard ratio (95% CI)			Incidence, %†	Hazard ratio (95% CI)	
		Univariate	Multivariate‡			Univariate	Multivariate‡
Non-Beijing	0.26 (356/1,396)	Referent	Referent		0.69 (845/1,236)	Referent	Referent
Beijing	0.39 (62/160)	1.53 (1.09–2.14)	1.65 (1.17–2.33)		0.73 (118/162)	1.02 (0.80–1.30)	1.03 (0.80–1.33)

*Likelihood ratio test for the interaction between age (<15 or >15 y) and index patient *M. tuberculosis* lineage: p = 0.048. †Per person-year for the univariate model. ‡Adjusted for index patient drug resistance profile; index patient age (16–30, 31–45, 46–60, and >60 y); index patient HIV status; household contact age (0–5, 6–10, 11–15, 16–30, 31–45, 46–60, and >60 y); household contact *M. bovis* BCG vaccination status; household contact socioeconomic status; household contact nutritional status.

### Progression to Active TB

**Figure 3:**
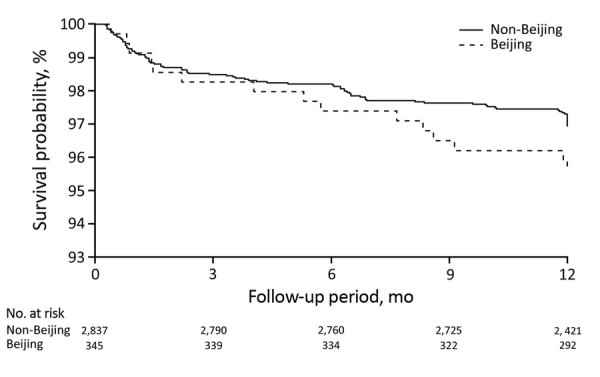
Survival curves for incident tuberculosis in child household contacts by index patient *Mycobacterium tuberculosis* lineage, Lima, Peru, September 2009–August 2012.

**Figure 4:**
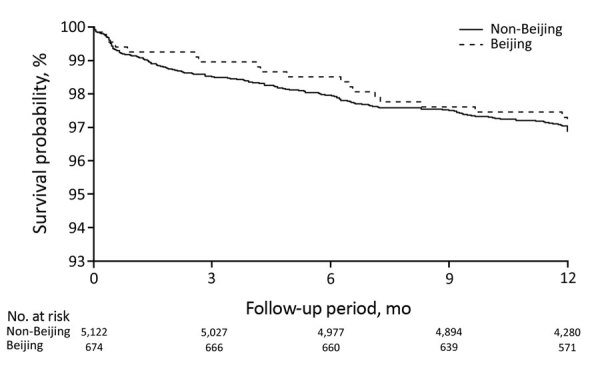
Survival curves for incident tuberculosis in adult household contacts by index patient *Mycobacterium tuberculosis* lineage, Lima, Peru, September 2009–August 2012.

After we adjusted for potential confounders, we found the HR for TB among child contacts exposed to an index patient with Beijing strains compared with non-Beijing strains to be 1.45 (95% CI 0.77–2.7). We found no evidence that exposure to Beijing strains affected the risk for incident TB in adult contacts (aHR 1.06 [95% CI 0.64–1.77]) (Table 6; Figures 3, 4). The results persisted when we restricted the analysis to household contacts in whom TB was diagnosed within >30 days after index patient diagnosis (Appendix Table 8).

**Table 6:** Hazard ratios of incident tuberculosis among contacts exposed to a *Mycobacterium tuberculosis* Beijing lineage and a non-Beijing lineage, Lima, Peru, September 2009–August 2012

Lineage	Age <15 y*					Age >15 y*		
	Incidence, %†		Hazard ratio (95% CI)			Incidence, %†	Hazard ratio (95% CI)	
			Univariate	Multivariate‡			Univariate	Multivariate‡
Non-Beijing		3,043 (84/2,760)	Referent	Referent		2,874 (156/4,993)	Referent	Referent
Beijing		4,478 (15/335)	1.42 (0.78–2.59)	1.45 (0.77–2.72)		3,124 (19/661)	0.93 (0.57–1.52)	1.06 (0.64–1.77)

*Likelihood ratio test for the interaction between age (<15 or >15 y) and index patient *M. tuberculosis* lineage: p = 0.231. †Per 100,000 person-years in the univariate model. ‡Adjusted for index patient drug resistance profile; index patient age (16–30, 31–45, 46–60, and >60 y); index patient HIV status; household contact age (0–5, 6–10, 11–15, 16–30, 31–45, 46–60, and >60 y); household contact *M. bovis* BCG vaccination status; household contact SES; household contact nutritional status; household contact use of isoniazid preventive therapy; household contact TB history.

### Effect of Index Patient *M. tuberculosis* Lineage on the Protective Efficacy of BCG Vaccine

Although they lacked statistical significance, the aRRs for TB outcomes were higher for BCG-vaccinated children exposed to Beijing strains than for non–BCG-vaccinated children exposed to Beijing strains (aRR 1.01 [95% CI 0.69–1.49] for *M. tuberculosis* infection at baseline; aRR 1.21 [95% CI 0.90–1.63] for infection by 12 months; aRR 1.84 [95% CI 0.77–4.39] for TST conversion; aRR 1.96 [95% CI 0.41–9.39] for incident TB). This finding raises the possibility that BCG vaccination might be less efficacious in children exposed to Beijing strains than in those exposed to other strains (Table 7). Among BCG-vaccinated adult household contacts exposed to Beijing strains, the aRRs were 1.00 (95% CI 0.78–1.29) for *M. tuberculosis* infection at baseline, 1.02 (95% CI 0.89–1.16) for infection by 12 months, 1.00 (95% CI 0.48–2.06) for TST conversion, and 0.53 (95% CI 0.17–1.68) for incident TB (Table 7).

**Table 7:** Effects of BCG vaccination on *M. tuberculosis* infection and TB outcomes by lineage exposure for child and adult household contacts of culture-positive pulmonary tuberculosis patients, Lima, Peru, September 2009–August 2012*

Category and BCG vaccination status	Non-Beijing lineage			Beijing lineage		p value
	Infection prevalence, no. (%)	RR (95% CI)		Infection prevalence, no. (%)	RR (95% CI)	
Children, <15 y						
Baseline *M. tuberculosis* infection, n = 2,949						0.691
Without BCG vaccination	137 (27)	Referent		19 (32)	Referent	
With BCG vaccination	552 (26)	0.94 (0.81–1.09)		83 (32)	1.01 (0.69–1.49)	
*M. tuberculosis* infection by 12 months of follow-up, n = 2,417						0.405
Without BCG vaccination	187 (45)	Referent		26 (50)	Referent	
With BCG vaccination	853 (50)	1.07 (0.97–1.19)		139 (60)	1.21 (0.90–1.63)	
	Incidence†	HR (95% CI)		Incidence†	HR (95% CI)	
Time to TST conversion						0.579
Without BCG vaccination	0.18 (48/260)	Referent		0.21 (7/32)	Referent	
With BCG vaccination	0.27 (292/1,065)	1.5 (1.08–2.08)		0.45 (53/119)	1.84 (0.77–4.39)	
Time to TB						0.168
Without BCG vaccination	4,585 (25/545)	Referent		3,098 (2/65)	Referent	
With BCG vaccination	2,619 (60/2291)	0.58 (0.35–0.96)		4,639 (13/280)	1.96 (0.41–9.39)	
	Infection prevalence, no. (%)	RR (95% CI)		Infection prevalence, no. (%)	RR (95% CI)	
Adults, >15 y						
Baseline *M. tuberculosis* infection, n = 5,381						0.611
Without BCG vaccination	260 (47)	Referent		27 (51)	Referent	
With BCG vaccination	2,263 (54)	1.07 (0.98–1.18)		313 (54)	1.00 (0.78–1.29)	
*M. tuberculosis* infection by 12 months of follow-up, n = 4,716						0.315
Without BCG vaccination	334 (72)	Referent		39 (81)	Referent	
With BCG vaccination	3,042 (82)	1.10 (1.04–1.16)		419 (82)	1.02 (0.89–1.16)	
	Incidence†	HR (95% CI)		Incidence†	HR (95% CI)	
Time to TST conversion						0.315
Without BCG vaccination	0.41 (70/172)	Referent		0.77 (10/13)	Referent	
With BCG vaccination	0.72 (728/1,020)	1.55 (1.18–2.04)		0.73 (105/144)	1.00 (0.48–2.06)	
Time to TB						0.732
Without BCG vaccination	4,813 (28/582)	Referent		6,574 (4/61)	Referent	
With BCG vaccination	2,860 (129/4511)	0.66 (0.43–1.02)		2,434 (15/616)	0.53 (0.17–1.68)	

*BCG, *M. bovis* BCG; HR, hazard ratio; RR, risk ratio; TB, tuberculosis; TST, tuberculin skin test. †Cases per person-year (no. converted/no. tested).

## Discussion

In our study, children exposed to strains of *M. tuberculosis* Beijing sublineage were more likely than were those exposed to strains of other lineages to become infected. In addition, children exposed to Beijing strains were more likely than children exposed to other strains to progress to TB, although the relative risk of disease progression was not statistically significant (type 1 error rate of 0.05). We did not observe these effects among adult contacts.

Our results are consistent with those from previous studies that assessed the relative transmissibility of the Beijing strain through cohort-based studies or molecular epidemiology. In a study in South Africa, Marais et al. found that children <5 years of age who had household exposure to Beijing strains were 1.5 times more likely than those exposed to other strains to be TST-positive at baseline (*13*). In a prospective cohort study of household contacts in The Gambia, de Jong et al. found that Beijing lineage–exposed contacts were 7 times more likely than those exposed to the *M. africanum* lineage to progress to TB after 2 years of follow-up but were equally likely to be TST-positive at baseline or convert at 3 months (*12*). In the De Jong et al. study, the proportion of TST-positive household contacts at baseline and the incidence of TST conversion and disease did not differ significantly between Beijing and other strains within *M. tuberculosis* sensu strictu.

Although some molecular epidemiologic studies have found that Beijing strains are more likely than other strains to form genotypic clusters, other studies have not supported this conclusion. Many of these studies assumed that patients within a chain of recent *M. tuberculosis* transmission will share molecular fingerprints (*32**–**36*) and that clustering of genotypes is therefore a proxy for transmissibility and disease progression. In China, Yang et al. reported that patients infected with Beijing strains were 1.56 times more likely than those infected with other lineages to share molecular fingerprints with other patients (*15*). Similar findings have been reported from Lima; in a study by Imawoto et al., 80% of Beijing strains were in clusters, although clustering among other strains was not reported (*14*). In Vietnam, Holt et al. reported that, although the Beijing strains accounted for 58% of the total study sample, they constituted 70% of all clustered strains (*21*). In Papua New Guinea, Bainomugisa et al. found that 82% of isolates with Beijing strains were found in clusters, compared with 43% of isolates with strains from other lineages (*37*). In China, Yang et al. reported that the proportions of clustering were 34% for Beijing and 18% for non-Beijing strains (*38*). However, in Canada, Langlois-Klassen et al. found that Beijing was less likely to be found in clusters than other strains (21% vs. 37%) (*16*), and in the Netherlands, Nebenzahl-Guimaraes et al. showed that Beijing strains were no more transmissible than strains of other lineages, after adjustment for host factors using an approach that controlled for the propensity of a strain to propagate (*17*).

Some animal studies have provided evidence to support the hypothesis that Beijing strains are more likely than other lineages to cause disease. Mice experimentally infected with Beijing strains not only died earlier and had higher death rates but also had more lung tissue damage than controls (*39**–**42*). Some in vitro studies of macrophages have also found that the Beijing strain can downregulate the expression of pathogen recognition receptors and major histocompatibility complex class II, modify the secretion of inflammatory cytokines, and induce the necrosis of host immune cells (*43**–**46*).

We considered possible explanations for the difference in the effect of the Beijing strain in children and adults. Given their mixing patterns, adults may be more likely than children to be infected within the community rather than in the household. If the household contacts in the cohort reported here had been infected by someone other than the household index patient, strain-specific exposure status might have been misclassified. Such misclassification would have been more likely in adults and would have biased the results for this group toward the null of no effect. However, another possibility is that Beijing strains might lead to earlier disease progression in younger persons with newly acquired *M. tuberculosis*. Several studies from Vietnam report that Beijing strains make up the highest proportion of incident cases in persons <20 years of age and that the prevalence of Beijing strains declines with increasing age (*18**,**19*). In Iran, Erie et al. similarly showed that 27% of patients <20 years of age were infected by Beijing strains and that the prevalence of Beijing strains among patients >20 years of age was 13% (*20*). The increasing prevalence of Beijing strains in children may be related to use of BCG vaccine. This explanation would be consistent with our finding that that the protective efficacy of BCG vaccine against Beijing strains was reduced in children but not in adults. Possible explanations for these observations include a decrease in the protective efficacy of BCG vaccination with increasing age, a shift in the administered BCG strain in Peru’s recent history, or a reduction in the immunogenicity of BCG vaccine over time (*47**–**49*).

Our study had several notable limitations. First, within a high-transmission setting such as Lima, children still could have been infected outside the household. Misclassification of the lineage exposure status in children also would have led to an underestimation of the effect. Second, discrepancies between 24-locus MIRU-VNTR genotyping and WGS have been noted previously (*50*), and some lineages assigned on the basis of MIRU-VNTR could have been inaccurate, again leading to a misclassification error that could have underestimated the true effect of Beijing strains.

In conclusion, we found that exposure to household index patients infected with Beijing strains was associated with increased risk for TST conversion and disease in children <15 years of age but not in adults. These findings raise the possibility that genotypic variation in *M. tuberculosis* may have important phenotypic effects that should be further studied. In particular, it will be essential to determine whether BCG vaccination provides less protection against Beijing strains than strains from other lineages and whether the efficacy of any newly developed vaccines varies by *M. tuberculosis* genoype.

Appendix Additional results in a study of Beijing lineage and risk for tuberculosis in child household contacts, Lima, Peru.
