# Supplementary material for: Mycobacterium tuberculosis Beijing Lineage and Risk for Tuberculosis in Child Household Contacts, Peru
Source: Emerg Infect Dis. 2020 Mar;26(3):568–78. doi: 10.3201/eid2603.191314 (PMC7045848; doi:10.3201/eid2603.191314)
Supplement: Appendix — Additional results in a study of Beijing lineage and risk for tuberculosis in child household contacts, Lima, Peru. [file 19-1314-Techapp-s1.pdf]

Article DOI: https://doi.org/10.3201/eid2603.191314

Mycobacterium tuberculosis Beijing Lineage and Risk for Tuberculosis in Child Household Contacts, Peru

Appendix

Appendix Table 1. Baseline characteristics of child household contacts by baseline *Mycobacterium tuberculosis* infection status

Variable	Uninfected, no. (%)*	Infected, no. (%)*	p value†
Age, y			
0–5	1,066 (47)	286 (34)	<0.001
6–10	644 (28)	262 (31)	
11–15	564 (25)	293 (35)	
Sex, M	1,159 (51)	407 (48)	0.211
HIV seropositive	3 (<1)	0	0.568
Self-reported diabetes	1 (<)	1 (<1)	0.465
Current smoker	2 (<1)	2 (<1)	0.296
M. tuberculosis lineage exposure			
L2 (Beijing)	223 (10)	110 (13)	<0.001
L4.1	912 (40)	382 (45)	
L4.3	891 (39)	272 (32)	
Other	248 (11)	77 (9)	
Presence of *M. bovis* BCG scar	1,847 (81)	675 (80)	0.538
Nutritional status			
Normal weight	1775 (79)	667 (80)	0.178
Underweight	71 (3)	16 (2)	
Overweight	399 (18)	151 (18)	
Socioeconomic status			
Low	846 (38)	350 (43)	0.038
Middle	1,012 (45)	336 (41)	
High	373 (17)	127 (16)	
Isoniazid preventive therapy recipient	1,193 (52)	437 (52)	0.809

*Total number varied because of missing data.
†χ-square test p value.

Appendix Table 2. Baseline characteristics of adult household contacts by baseline *Mycobacterium tuberculosis* infection status

Variable	Uninfected, no. (%)*	Infected, no. (%)*	p value†
Age, y			
16–30	1,263 (48)	1,097 (36)	0.001
31–45	682 (26)	871 (29)	
46–60	413 (16)	717 (24)	
>60	298 (11)	322 (11)	
Sex, M	1,122 (42)	1,288 (43)	0.674
HIV seropositive	7 (<1)	19 (1)	0.067
Self-reported diabetes	63 (2)	93 (3)	0.11
Current smoker	219 (8)	304 (10)	0.015
M. tuberculosis lineage exposure			
L2 (Beijing)	309 (12)	353 (12)	0.574
L4.1	1,050 (40)	1,240 (41)	
L4.3	1,015 (38)	1,108 (37)	
Other	282 (11)	306 (10)	
Presence of *M. bovis* BCG scar	2,321 (87)	2,704 (90)	0.003
Nutritional status			
Normal weight	1,337 (51)	1,276 (43)	0.001
Underweight	23 (1)	42 (1)	
Overweight	1,272 (48)	1,664 (56)	
Socioeconomic status			
Low	781 (30)	1,001 (34)	0.007
Middle	1,214 (47)	1,289 (44)	
High	594 (23)	646 (22)	
Isoniazid preventive therapy recipient	224 (8)	169 (6)	0.001

*Total number varied because of missing data.
†χ-square test p value.

Appendix Table 3. Baseline characteristics of child household contacts by incident tuberculosis status

Variable	Disease free, no. (%)*	Secondary tuberculosis, no. (%)*	p value†
Age, y			
0–5	1,350 (44)	57 (40)	0.027
6–10	898 (29)	33 (33)	
11–15	834 (27)	53 (37)	
Sex, M	1,545 (50)	75 (52)	0.648
HIV seropositive	3 (<1)	0	0.999
Self-reported diabetes	2 (<1)	0	0.999
Current smoker	4 (<1)	0	0.999
Presence of *Mycobacterium bovis* BCG scar	2,498 (81)	112 (87)	0.477
Nutritional status			
Normal weight	2,404 (79)	123 (87)	0.081
Underweight	93 (3)	2 (1)	
Overweight	548 (18)	17 (12)	
Socioeconomic status			
Low	1,192 (40)	61 (44)	0.604
Middle	1,325 (44)	59 (42)	
High	494 (16)	20 (14)	
Isoniazid preventive therapy recipient	1,641 (53)	40 (28)	<0.001

*Total number varied because of missing data.
†χ-square test p value.

Appendix Table 4. Baseline characteristics of adult household contacts by incident tuberculosis status

Variable	Disease free, no. (%)*	Secondary tuberculosis, no. (%)*	p value†
Age, y			
16–30	2,304 (41)	153 (53)	0.001
31–45	1,560 (28)	61 (21)	
46–60	1,137 (20)	46 (16)	
>60	619 (11)	30 (10)	
Sex, M	2,356 (42)	159 (55)	<0.001
HIV seropositive	26 (<1)	4 (1)	0.086
Self-reported diabetes	153 (3)	9 (3)	0.838
Current smoker	524 (9)	26 (9)	0.91
Presence of *Mycobacterium bovis* BCG scar	4,991(89)	247 (85)	0.067
Nutritional status			
Normal weight	2,536 (46)	190 (66)	<0.001
Underweight	55 (1)	13 (5)	
Overweight	2,979 (53)	84 (29)	
Socioeconomic status			
Low	1,749 (32)	106 (37)	0.117
Middle	2,473 (45)	122 (43)	
High	1,259 (23)	55 (19)	
Isoniazid preventive therapy recipient	403 (7)	6 (2)	<0.001

*Total number varied because of missing data.
†χ-square test p value.

Appendix Table 5. Effect of the *Mycobacterium tuberculosis* Beijing lineage on the risk for tuberculin skin test positivity at baseline in child and adult household contacts*

	Age ≤15 y, n = 2,981			**Age >15 y, n = 4,968**		
		**Rate ratio (95% CI)**			**Rate ratio (95%CI)**	
Lineage	**Prevalence of infection, no. (%)†**	**Univariate**	**Multivariate‡**	**Prevalence of infection, no. (%)†**	**Univariate**	**Multivariate‡**
Non-Beijing	619 (23)	Referent	Referent	2,022 (46)	Referent	Referent
Beijing	88 (28)	1.27 (1.06–1.53)	1.22 (0.99–1.52)	290 (48)	1.03 (0.94–1.14)	1.03 (0.93–1.14)

Excludes household contacts with history of tuberculosis, history of previous positive tuberculin skin test, or history of tuberculosis treatment.
†Prevalence of baseline *M. tuberculosis* infection univariate model.
‡Multivariate model adjusted for index patient drug resistance profile; index patient age (16–30, 31–45, 46–60, and >60 y); index patient HIV status; household contact age (0–5, 6–10, 11–15, 16–30, 31–45, 46–60, and >60 y); household contact *M. bovis* BCG status; household contact socioeconomic status; household contact nutritional status.

Appendix Table 6. Effect of the *Mycobacterium tuberculosis* Beijing lineage on the risk for tuberculin skin test positivity by 12 months of follow-up in child and adult household contacts*

	**Age ≤5 y, n = 2,387**			**>15 Years (n = 4,716)**		
		**Risk ratio (95% CI)**			**Risk ratio (95% CI)**	
Lineage	**Prevalence of infection, no. (%)†**	**Univariate**	**Multivariate‡**	**Prevalence of infection, no. (%)†**	**Univariate**	**Multivariate‡**
Non-Beijing	986 (47)	Referent	Referent	2,914 (79)	Referent	Referent
Beijing	88 (56)	1.21 (1.07–1.37)	1.23 (1.08–1.41)	411 (80)	1.03 (0.94–1.14)	1.02 (0.97–1.08)

*Excludes household contacts with history of tuberculosis, history of previous positive tuberculin skin test, or history of tuberculosis.
†Prevalence of *M. tuberculosis* infection at 12 months for the univariate model.
‡Multivariate model adjusted for index patient drug resistance profile; index patient age (16–30, 31–45, 46–60, and >60 y); index patient HIV status; household contact age (0–5, 6–10, 11–15, 16–30, 31–45, 46–60, and >60 y); household contact *M. bovis* BCG status; household contact socioeconomic status; household contact nutritional status.

Appendix Table 7. Comparison of hazard ratios for tuberculin skin test conversion between household contacts exposed to *Mycobacterium tuberculosis* Beijing lineage and non-Beijing lineage*

	Age ≤5 y			Age >15 y		
		Hazard ratio (95% CI)			Hazard ratio (95% CI)	
Lineage	Incidence†	Univariate	Multivariate‡	Incidence†	Univariate	Multivariate‡
Non-Beijing	0.32 (370/1,162)	Referent	Referent	0.69 (749/1,084)	Referent	Referent
Beijing	0.47 (62/132)	1.46 (1.05–2.02)	1.63 (1.16–2.28)	0.73 (105/142)	1.04 (0.83–1.32	1.07 (0.84–1.36)

*>6 mm increment in tuberculin skin test induration size.
†Cases per person-year for the univariate model.
‡Multivariate model adjusted for index patient drug resistance profile; index patient age (16–30, 31–45, 46–60, and >60 y); index patient HIV status; household contact age (0–5, 6–10, 11–15, 16–30, 31–45, 46–60, and >60 y); household contact *M. bovis* BCG status; household contact socioeconomic status; household contact nutritional status.

Appendix Table 8. Comparison of hazard ratios for incident tuberculosis* between contacts exposed to *Mycobacterium tuberculosis* Beijing lineage and non-Beijing lineage

	Age ≤15 y			Age >15 y		
		Hazard ratio (95% CI)			Hazard ratio (95% CI)	
Lineage	**Incidence†**	**Univariate**	**Multivariate‡**	**Incidence†**	**Univariate**	**Multivariate‡**
Non-Beijing	2,151 (61/2,836)	Referent	Referent	2,264 (113/4,991)	Referent	Referent
Beijing	3,478 (12/345)	1.57 (0.41–6.00)	1.48 (0.74–2.95)	2,118 (14/661)	0.92 (0.52–1.65)	1.02 (0.56–1.88)

*Tuberculosis diagnosed ≥30 days after the diagnosis in the index patient.
†Cases per 100,000 person-year for the univariate model.
‡Multivariate model adjusted for index patient drug resistance profile; index patient age (16–30, 31–45, 46–60, and >60 y); index patient HIV status; household contact age (0–5, 6–10, 11–15, 16–30, 31–45, 46–60, and >60 y); household contact *M. bovis* BCG status; household contact socioeconomic status; household contact nutritional status; household contact use of Isoniazid preventive therapy; household contact tuberculosis history.

Appendix Table 9. *Mycobacterium bovis* BCG status among household contacts of culture-positive index patients, by nutritional status and socioeconomic status

Variable	Non-BCG recipients, no. (%)	BCG recipients, no. (%)	p value*
Child contacts			
Nutritional status			
Normal weight	488 (81)	2,038 (79)	0.536
Underweight	16 (3)	79 (3)	
Overweight	99 (16)	466 (18)	
Socioeconomic status			
Low	241 (40)	1,012 (40)	0.237
Middle	273 (46)	1,111 (44)	
High	84 (14)	430 (17)	
Adult contacts			
Nutritional status			
Normal weight	385 (58)	2,348 (45)	<0.001
Underweight	16 (2)	52 (1)	
Overweight	265 (40)	2,805 (54)	
Socioeconomic status			
Low	208 (32)	1,649 (32)	0.6
Middle	304 (47)	2,303 (45)	
High	139 (21)	1,174 (23)	

*χ-square test p value.
